# From *In Vivo* to *In Vitro*: Dynamic Analysis of *Plasmodium falciparum var* Gene Expression Patterns of Patient Isolates during Adaptation to Culture

**DOI:** 10.1371/journal.pone.0020591

**Published:** 2011-06-06

**Authors:** Qingfeng Zhang, Yilong Zhang, Yufu Huang, Xiangyang Xue, He Yan, Xiaodong Sun, Jian Wang, Thomas F. McCutchan, Weiqing Pan

**Affiliations:** 1 Institute of Infectious Disease and Vaccine Development, Tongji University School of Medicine, Shanghai, China; 2 Department of Pathogen Biology, Second Military Medical University, Shanghai, China; 3 Yunnan Institute of Parasitic Diseases, Puer, China; University of Copenhagen, Denmark

## Abstract

*Plasmodium falciparum* erythrocyte membrane protein 1 (PfEMP1), encoded by the *var* gene family, plays a crucial role in disease virulence through its involvement in binding to various host cellular receptors during infection. Growing evidence suggests that differential expression of the various *var* subgroups may be involved in parasite virulence. To further explore this issue, we have collected isolates from symptomatic patients in south China-Myanmar border, and characterized their sequence diversity and transcription profiles over time of *var* gene family, and cytoadherence properties from the time of their initial collection and extending through a two month period of adaptation to culture. Initially, we established a highly diverse, DBLα (4 cysteines) subtype-enriched, but unique local repertoire of *var*-DBL1α sequences by cDNA cloning and sequencing. Next we observed a rapid transcriptional decline of *upsA*- and *upsB*-subtype *var* genes at ring stage through qRT-PCR assays, and a switching event from initial ICAM-I binding to the CD36-binding activity during the first week of adaptive cultivation *in vitro*. Moreover, predominant transcription of *upsA var* genes was observed to be correlated with those isolates that showed a higher parasitemia at the time of collection and the ICAM-1-binding phenotype in culture. Taken together, these data indicate that the initial stage of adaptive process *in vitro* significantly influences the transcription of virulence-related *var* subtypes and expression of PfEMP1 variants. Further, the specific upregulation of the *upsA var* genes is likely linked to the rapid propagation of the parasite during natural infection due to the A-type PfEMP1 variant-mediated growth advantages.

## Introduction

*Plasmodium falciparum* is the most virulent species of malaria that infects humans. Erythrocytes infected with this parasite adhere to different types of endothelial cells in the deep vasculature of the body to avoid being cleared by the spleen [1], [2]. The specificity of adherence is mediated by a polymorphic protein, *P. falciparum* erythrocyte membrane protein 1 (PfEMP1), encoded by members of a multi-gene family (the *var* genes) [3], [4]. It is generally thought that only one member of this family is selectively expressed each generation and migrates to the surface of the infected RBC dictating the cellular receptor to which the erythrocyte will bind [5], [6]. There are approximately 60 copies of the PfEMP1 encoded in each genome of 3D7 strain [7], [8]. The rate of change in expression may reach ∼18% per generation *in vivo* which serves to compromise the immune defense of the host [9], [10]. The large, highly variable protein includes several sequence motifs such as the various Duffy-binding-like domains (DBL) and the cysteine rich inter-domain regions (CIDR). All interact with various host cellular receptors such as CD36, ICAM-1, CSA, E-Selectin, VCAM, etc. [1], [11], [12].

*Var* gene family can be separated into three major groups based upon conserved sequence features (A, B and C) upstream of the protein coding region [13]. Several studies have investigated the potential association between PfEMP1 expression and presentation of malaria [14], [15], [16], [17], [18]. It has been suggested, on the basis of studies conducted in Tanzania and Papua New Guinea, that expression of group A or B/A *var* genes are associated with severe children malaria, while group C *var* genes are linked to asymptomatic malaria. In support, large-scale sequence analysis of *var*-DBL1α domains revealed that the lack of 1 or 2 cysteines in this region, most belonging to group A or B/A *var* genes, was associated with severe malaria [19], [20], [21]. Although these investigations indicate that the specific expression of subtype *var* genes play a crucial role in the pathogenesis of different forms of malaria, little is known of the underlying mechanism.

The individual clonal type of *P. falciparum* contains approximately 60 copies of this gene family, and the collection of clonal types of field isolates that constitute the global population of this parasite seems to be nearly endless. Nevertheless, studies on geographically diverse populations support the existence of structured *var* repertoires among various geographic areas, although their sequences are highly diverse within each repertoire [19], [22], [23], [24]. These investigations have established several *var* repertoires of field isolates in Africa and South America, however, little is known of the parasites in southern China-Myanmar border, a highly epidemic area in Asia [25], [26].

In a clonal population the most abundant transcript(s) found in each blood-stage cycle is thought to be expressed and therefore reflective of adherent specificity during *in vitro* cultivation. For that reason, much of the information we have about *var* genes comes from transcriptional data. The direct investigation of protein properties would be ideal, but most often such experiments are not done due to the degree of difficulty involved in studying each member and the high number of molecules involved. Further, the study of PfEMP1 *in vitro* is potentially impacted by artifacts relating both to the effects on the parasite of extended periods in culture and the technical necessity to equate RNA expression with protein presence and hence function. Peters et al have shown such effect on *var* gene transcription of experimentally infected 3D7 strain during adaptive cultivation [27]. Hence it is important to understand the effects of *in vitro* adaptation of patient isolates to laboratory populations where the dominant selective forces are different than those affecting the parasite in its native state.

In this study, we investigated the composition of *var* gene transcripts, their transcription patterns during the life cycle of the parasite, and their cytoadherent characteristics from *in vivo* to *in vitro* over the course of ∼2 months. In so doing we hoped to clarify the influence of the adaptive process on the transcription as well as expression of *var* genes, and look for potential link between PfEMP1 expression and parasite characteristics starting from each isolate's collection point, and extending through many generations of *in vitro* culture. Our data revealed a potential correlation between the predominant transcription of *upsA*-subtype *var* genes and the parasitemia of parasites found in the blood when the isolates were collected, which partially reflected the growth rate of parasite *in vivo*. We therefore suggest that A-type PfEMP1 variants-mediated high parasitemia contributes to the virulence of parasites in natural infection.

## Results

### Distribution of the conserved cysteine/PoLV motifs in DBL1α sequences

**Figure 1:**
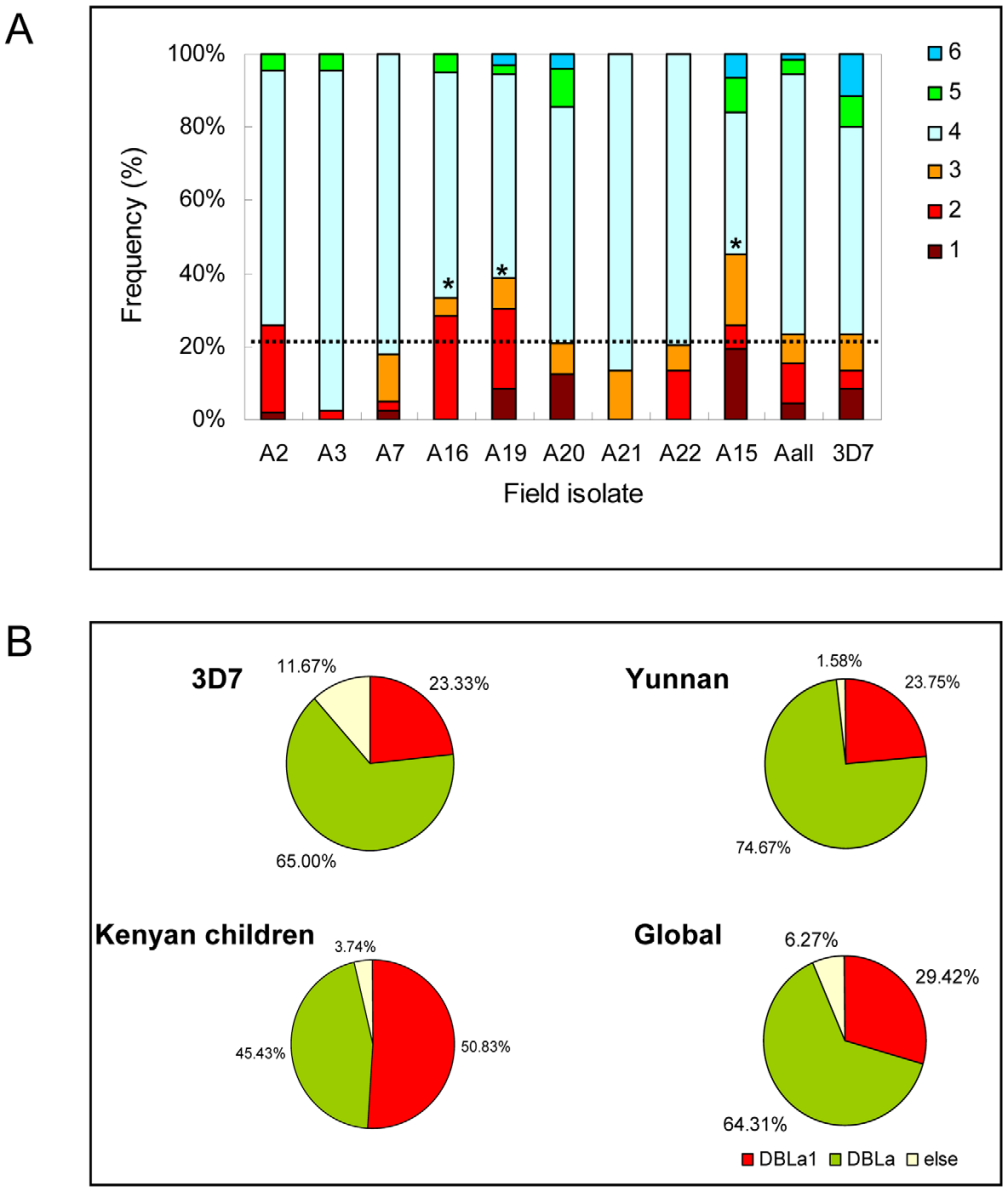
Classification of *var*-DBL1α transcript sequences of Yunnan isolates in comparison with other field isolates and 3D7 strain. (A) DBL1α amino acid sequences derived from cDNA were divided into six sequence groups in terms of the conserved “cysteine/PoLV” sequence tag within the DBL1α region. The distribution of different subsets of individual isolate and overall cDNA sequences (A_all_) of the 9 Yunnan isolates were shown in each bar with 3D7 strain (genomic sequence) as reference. The 95% confidence values (*p*) for the *χ* ^2^ test between DBLα1 (subgroup 1∼3) and others was: *p*<0.01*. (B) The distribution of each subgroups of DBL1α sequence (cDNA) based on the number of cysteines in Yunnan symptomatic malaria isolates were analyzed with 3D7 (gDNA), Africa (cDNA) and global sequences (cDNA) as references. The subgroup *var* genes were labeled as follows: red, cys2 (DBLα1); green, cys4 (DBLα); white, cysX (else).

Initially, we analyzed the *var* transcripts from 9 clinical isolates collected from symptomatic malaria adult patients with multi-infection history in the Yunnan province of south China (Table 1 and S1), and compared them with previously reported isolates collected from other regions of the world. It has been shown that all DBL1α sequences can be grouped into 6 subgroups according to the cysteine/PoLV structural features within this DBL1α region of transcripts [19], [20]. In laboratory 3D7 strain, *UpsA var* transcripts fall into the 1–3 subgroups while most *upsB* and *upsC var* genes belong to the 4–6 subgroups. Analysis of our isolates showed that A15, A16 and A19 had a higher proportions of transcripts fitting into the 1–3 subgroups than other samples (p<0.01). In contrast, the overall distribution of these motifs in *var* transcripts was similar to that of non-severe malaria cases found elsewhere [28], and that in the genome of 3D7 strain (Figure 1A).

**Table 1:** Clinical assessment of symptomatic malaria patients recruited in this study.

Isolates	Age(ys)/ gender	Strain (stage)	Multi- infection	Parasitemia (/µl blood)	Symptom	Dominant *var* transcripts (*ups*)^ d^
A2^a^	50/M	Pf (R)	1	1620	fever, headache	B, C ^1^
A3^a^	24/M	Pf (R)	2	9120	fever, headache	B, C ^1^
A7^a^	34/M	Pf (R)	1	36300	fever, systemic ache	B, C ^1^
A15^a^	37/M	Pf (R)	3	209400	fever	A, B ^1^
A16^a^	38/M	Pf (R)	2	194400	fever, headache	A, C ^1^
A19^a^	36/M	Pf (R)	2	122100	fever	A, B, C ^1^
A20^a^	35/M	Pf (R)	2	7500	fever, headache	B, C ^1^
A21^a^	34/M	Pf (R)	2	11280	fever, systemic ache	B, C ^1^
A22^a^	42/M	Pf (R)	2	8580	fever, systemic ache	C ^1^
F07-4^b^	14/M	Pf (R)	1	38157	fever	ND^2^
F08B-5^b^	18/F	Pf (R)	1	186667	fever, headache	ND
F08B-9^b^	25/F	Pf (R)	2	10952	fever	ND
F08B-33^b^	16/M	Pf (R)	1	10099	fever, headache	ND
F08B-34^ b^	44/F	Pf (R)	2	975	fever	ND
LZF22^b^	26/F	Pf (R)	2	2068	fever	ND
LZF25^b^	29/M	Pf (R)	1	17950	fever, headache	ND
LZF26^b^	19/F	Pf (R)	1	3980	fever	ND
YN3^c^	32/M	Pf (R)	1	1733360	ague, coma	A, B, BC ^3^
YN8^c^	47/M	Pf (R)	1	10320	fever, ague	B, BC ^3^
YN11^c^	17/M	Pf (R)	2	22160	fever, ague	BC ^3^
YN27^c^	6/M	Pf (R)	2	131200	fever, ague	BC ^3^
YN29^c^	16/M	Pf (R)	1	273760	fever, ague, coma	BC ^3^
YN53^c^	18/M	Pf (R)	1	620000	fever, ague	BC ^3^

a.Total RNA extracted form Yunnan field isolates were used in diversity and distribution analysis of *var*-DBLα sequence at the time point of collection (“0” hr).

b.Field isolates from Yunnan and Myanmar used in adaptive cultivation *in vitro* for ∼2 month, and measured of transcription profile and cytoadherent features during the course.

c.Myanmar field isolates used in adaptive cultivation *in vitro* for ∼16 days, and measured of transcription profile in rings and trophozoites.

d.The dominant *var* transcript at collection time.

1.The dominant *var* transcripts were identified by cDNA cloning-sequencing strategy.

2.Not done.

3.The dominant *var* transcripts were identified by qRT-PCR with *var* subtype-specific primers.

**Figure 2:**
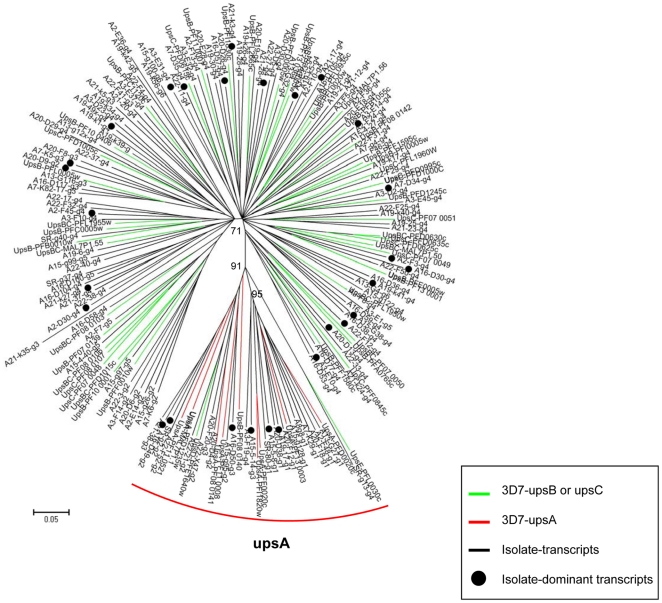
Phylogenetic trees of all *var*-DBL1α-contig amino acid sequences of Yunnan isolates. The unrooted radial trees were generated using the p-distance/NJ method as in Figure 1. Studied sequences were labeled as follows: green lines, *upsB* or *upsC var* genes of 3D7; red lines, *upsA var* genes of 3D7; black lines, *var*-DBL1α amino acid sequences of Yunnan field isolates cloned from cDNA. The dominantly transcribed *var* genes in individual field isolates were labeled as black circles. The labeling of the studied sequences was as follows: (1) the first number referred to the individual field isolates; (2) the *ups* “A,” “B,” or “C” indicated the *var* subgroups of 3D7 line. The 60 *var* genes of 3D7 strain (gDNA) were used as references.

According to the number of cysteine residues within the DBL1α domains, the 1–3 subgroups with two cysteines are also referred to as DBLα1 subtype, which has been shown to be associated with high virulence of parasites from field isolates, whereas the 4–5 subgroups with four cysteines belong to the DBLα subtype [28]. Here, we also analyzed the composition of transcript sequences from Yunnan isolates in terms of the summarized classification and compared them to that found in other studies [19], [20]. As shown in Figure 1B, Yunnan samples had a similar proportion of DBLα1 transcripts to that found in 3D7 genome, but significantly lower than that reported for field isolates from African children as well as the global samples (*p*<0.01). It is worth noting that the African children analyzed here were generally severe malaria patients including cerebral malaria, whereas the 3D7 clonal type has been adapted for cultivation condition *in vitro* for numerous generations. This result further demonstrates that the specific transcription of *var* subgroup with 4 cysteines in their DBL1α domain is associated with non-severe malaria under selection of host immune pressure. Further, a phylogenetic analysis with all of the DBL1α tags from Yunnan isolates revealed a unique cluster corresponding to *upsA var* genes in 3D7 strain. (Figure 2).

Finally, all DBL1α sequences of the Yunnan isolates were designated in terms of the nomenclature with distinct sequence tags as “PoLV1-PoLV2-PoLV3-(cys)n-PoLV4-length” [28]. In total of 130 *var*-DBL1α-contigs obtained from the 9 isolates described above, 93 sequences were unique in Yunnan area compared with those of geographic field isolates published elsewhere, suggesting a restricted local repertoire in this area (Table S2) [19], [20], [22].

### *Var* gene distribution in clinical isolates

**Figure 3:**
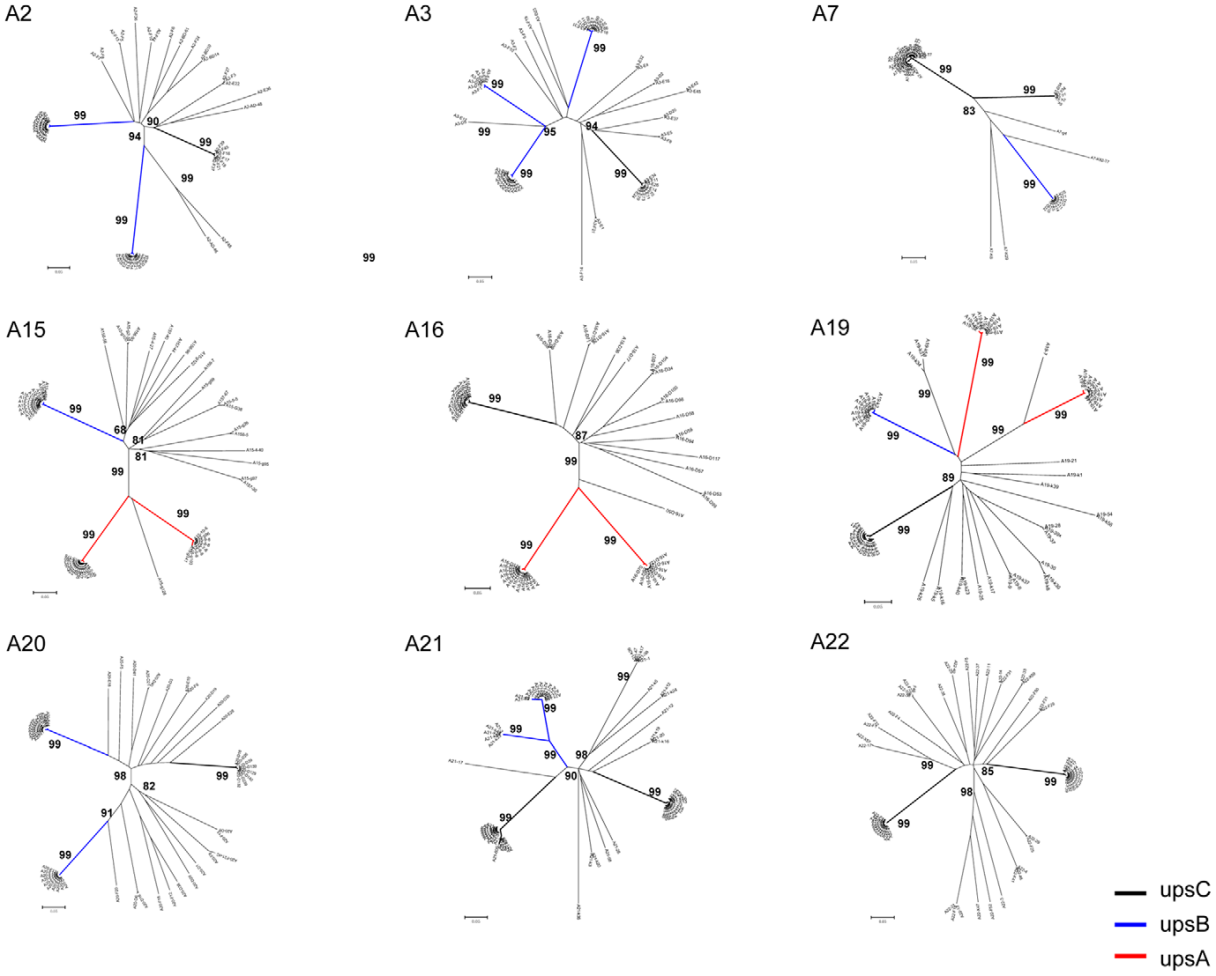
Phylogenetic trees of the *var*-DBL1α amino acid sequences (cDNA) from individual Yunnan isolate. The unrooted radial trees were generated using the p-distance/NJ method (Numbers at the nodes represented bootstrap proportions (BP) on 1000 replicates, and bootstraps above 60% were indicated). The scale bar represented the proportion of different amino acid compared. The *var*-DBL1α type with a copy number of >10 was referred as “dominant transcript” in each isolate, which was labeled by bold line with different colours: red, upsA; blue, upsB; and black, upsC, respectively.

To measure the infection complexity of each isolate, we determined the number of genotypes per isolate using a single copy gene, the Merozoite Surface Protein 2 (*MSP-2*), by PCR-RFLP analysis as described previously [29]. There was a mean of 1.9 infecting clones per isolate indicating a relatively low number of multiple infections of isolates in the area (Table 1). We then analyzed the transcript diversity within each of our isolates. In 9 out of 15 clinical isolates analyzed in this experiment, we obtained more than 50 *var*-DBL1α cDNA sequence reads per sample by sequencing of clones (Table 1 and S1). Isolates had a mean of 14.4 different DBL1α transcripts per sample of which only 2∼3 were representative of abundant transcripts when they had >10 copies (Figure 3). In agreement with similar studies this suggests 1∼2 dominant *var* gene transcripts per generation [30] and is consistent with our estimate of genotypes per isolate. The variety of expression profiles raised the possibility that the dosage of *var* transcription was being regulated at multiple sites during the course of development.

Among these samples, interestingly, three isolates with higher parasitemia, i.e. A15, A16 and A19, exclusively transcribed dominant *upsA* subgroup *var*s (*p*<0.01) (Figure 3 and Table 1), while the *var*-DBL1α sequences cloned from their genomic DNAs did not indicate a higher proportion of *upsA var* genes (data not shown). Isolates with *upsB* or *upsC* dominant *var* transcripts exhibited much lower parasitemia in peripheral blood.

### Monitoring the *var* gene family of isolates over the course of cultivation *in vitro*

**Figure 4:**
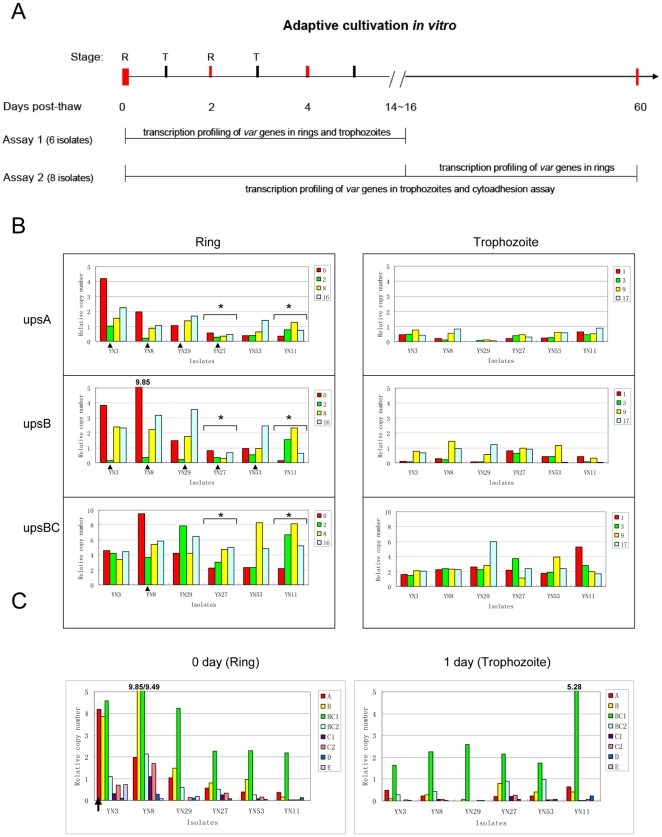
Transcription profile of subtype *var* genes in six isolates during the initial cultivation. (A) Two assays were designed according to the requirement of the material amount needed for different analysis. R, ring; T. trophozoite.(B) Transcriptional level of three major subtypes of *var* genes respectively in six field isolates cultivated *in vitro* for 17 days measured by qRT-PCR. The time points were shown as days post thawing, and those time points when the transcription level were significantly reduced were indicated with arrows. “*”: the isolates with multi-subclone at 0 hr and one of them had taken over the culture after thawing *in vitro*.(C) The transcription profiles of all subtype *var* genes of six field isolates during the first cycle in culture. The time point of the dominantly transcribed *upsA var*s were indicated by arrow.

In the work described above, we observed a potential link between the dominant *upsA var* transcripts and higher density of ring-stage parasites in peripheral blood. This raises the question of whether the transcription status of *upsA* and other *var*s are constant during adaptation to culture. Previously it has been reported that the overall abundance of *upsA*, -*D* and -*E* transcripts of *ex vivo* samples declined dramatically after a short period (the first ∼10 days) of *in vitro* cultivation [27]. This indicated that a selective process specifically affects those subgroups during parasite cultivation. To clarify these issues, we monitored the transcription patterns of *var* gene families at both ring and trophozoite stages of individual isolates over a two month period of in vitro cultivation through quantitative reverse transcription PCR (qRT-PCR) with *var* subtype-specific primers. Due to the limited material of parasites in the initial stage during cultivation, two assays were designed for different analysis strategies. Six isolates were cultivated for 14∼16 days and entire culture were used to monitor *var* transcription at both ring and trophozoite stages of development, whereas the other eight isolates were maintained in culture for up to ∼2 months while monitoring both adhesive properties and trophozoite-stage transcription over the entire period (Figure 4A, Table 1). Among these samples, all isolates had adapted to *in vitro* cultivation conditions within the first ∼15 days, nevertheless, the growth rates of individual isolates varied during the process. Therefore the sampling time points varied as indicated in the figures.

**Figure 5:**
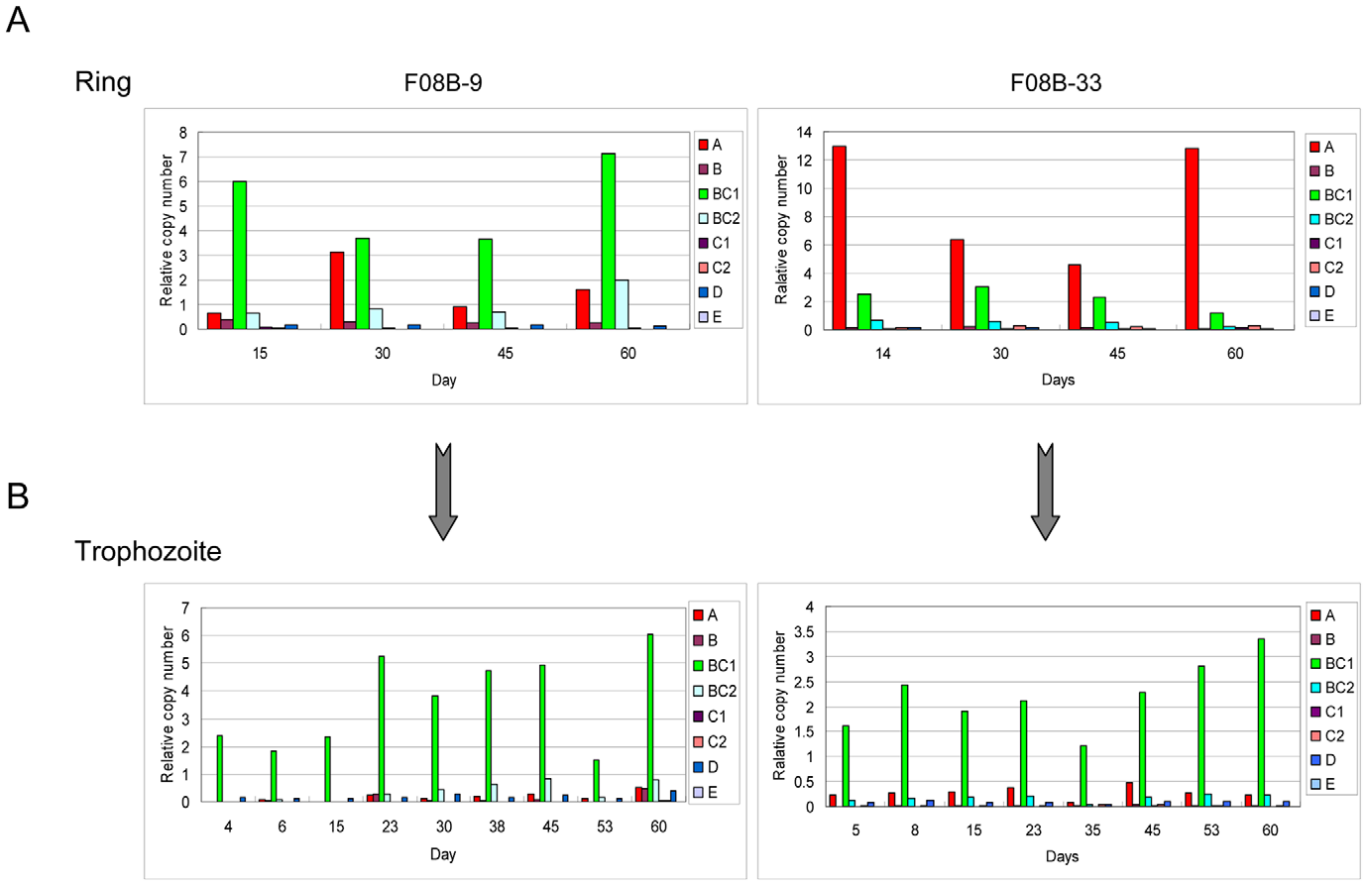
Transcription profile of subtype *var* genes at different stages in individual cycles. The ring-stage (A) and trophozoite-stage (B) transcription patterns of the *var* gene family of two representative isolates (F03B-9 and F03B-33) in culture for ∼2 months. The *Seryl-tRNA synthetase* gene was used as the endogenous control.

In most isolates, three subtype *var* genes, *upsA*, *upsB* and *upsBC1*, produced the most abundant transcripts at ring stage, while *upsBC1* subtype transcription alone dominated in trophozoite-stage parasites during the period of adaptive cultivation *in vitro* (Figure 4 and 5). It was notable that the only dramatic change in the transcription profile was a reduction in *upsA*- and *upsB*-subtype *var* transcripts at the initial stage, but they returned by the fourth cycle (Figure 4B). To avoid the probable bias coming from the change of sub-clonal proportion of each isolate due to their different growth advantages during the adaptive process, we measured the composition of sub-clones for the 6 isolates at various time points *in vitro* in comparison with that of the collection time by genotyping with microsatellite markers [31], [32]. Result showed that three isolates (YN3, YN8, YN29) with significant transcriptional decline of *upsA-* and *upsB- vars* had only a single clone and maintained the same one from *in vivo* to *in vitro* in two weeks. While a selection effect was observed in another 2 isolates (YN11 and YN27), in which one sub-clone had taken over the culture *in vitro*, no significant decrease of the two above-mentioned subtypes had occurred in these samples (Figure 4B and Table S3). Therefore, it implies that the first several developmental cycles are crucial to the adaptive process of parasites from field isolates, and that the transcriptional fluctuations of the two *var-*subtypes are reflective of this process.

In ring-stage parasites, two different transcription patterns of *var* gene family were observed. 12 out of 14 field isolates exhibited dominant transcripts of *upsBC1* subtype, whereas two isolate, i.e. YN3 and F08B-33, showed that the *uspA* subgroup was dominantly transcribed in rings (Figure 4B and 5A). All *var* genes were transcribed at much lower levels in trophozoites than in ring stage parasites except the *upsBC1* subgroup, which was exclusively transcribed in most cultures with a similar level to that in rings. In contrast to that in ring-stage parasites, the *upsBC1*-subtype transcripts in trophozoite stage remained the dominant and constant over the course of *in vitro* culture (Figure 5B). Due to the exon 2-specific amplification by the degenerated *upsBC1* primers, the dominant transcripts of late-stage parasites are expected to be derived from the intron promoter of *var* genes [33].

In addition, the transcription profiling of the *var* gene family in the first cycle *in vitro* revealed that only isolate YN3 had a higher transcriptional abundance of the *upsA*-subtype *var*s compared with other members during the ring stage (Figure 4C). It is notable because this isolate exhibited a relatively higher parasitemia at the collection time (Table 1). This phenomenon further supports the correlation between the specific *upsA*-subtype *var* transcription with the increased parasitemia *in vivo*.

### Cytoadhesion feature of isolates during cultivation *in vitro*

**Figure 6:**
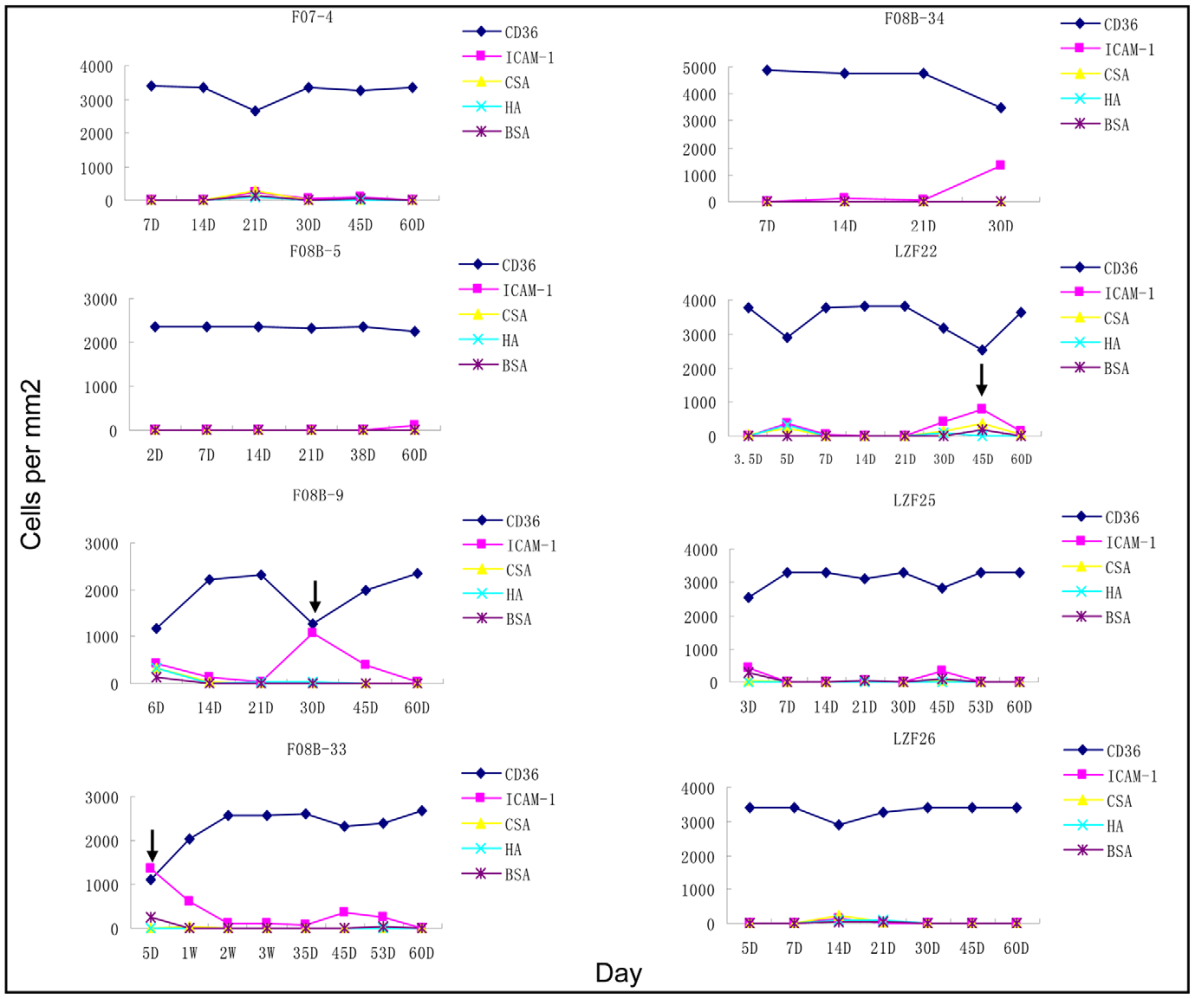
Dynamic analysis of cytoadherence features of eight isolates during the adaptive cultivation. Significant binding was defined as >5PRBC/mm2, and the final value of bound cells numbers was adjusted in terms of parasitemia of different batches used in the assay. The four purified receptors, CD36, ICAM-1, CSA, and HA were used as immobilized receptors with BSA as negative control. The time points of upregulation of ICAM-1 binding activity were indicated by arrows.

Finally, we tried to investigate the cytoadherence properties of each isolate for interaction with host cellular receptors such as CD36, ICAM-1, CSA and HA for a period from the point of collection and extending to the first two months of adaptation. Initially, we attempted to establish the association between transcription of specific *var* subtypes and cytoadhesion phenotypes in the laboratory FCC1/HN strain [34]. In this unselected culture, the link between *upsBC* and CD36 or *Var2CSA* and CSA phenotype were observed (Figure S1). The first has been described by Kraemer and Robinson [35], [36], and the second association was well studied with parasites involved in pregnancy-associated malaria (PAM) [37], [38]. In this study, CD36 was the main cytoadhesion feature in all cultivated isolates except the initial stage of F08B-33, and the CD36-binding property was retained over the two months *in vitro* (Figure 6).

The cytoadhesive pattens of various receptors used here were relatively constant during the cultivation in vitro, except a few time points. ICAM-1 binding was detectible at a few time points for isolates F08B-9 (30 days), F08B-34 (30 days) and LZF22 (45 days). Interestingly, isolate F08B-33 bound to ICAM-1 at the start of the study and then switched to CD36 over the first week in culture. Combined with the transcription analysis of these isolates corresponding to these timepoints, we observed a potential link between the ICAM-1 binding phenotype and dominant transcription of *upsA*-subtype *var* genes (Figure 5A and S2).

## Discussion

The worldwide high diversity of *var* primary sequences through frequent recombination among field strains, and the switching expression of *var* genes in different generations contribute to the complexity of the malaria pathogen [19], [22], [24], [39], [40], [41]. To explore the sequence polymorphism and its association with different malaria symptoms, the *var* gene sequences have been extensively investigated in terms of conserved features of functional regions as DBL1α domain in highly epidemic area in Africa or South America [19], [20], [28]. However, little is known of the filed isolates in southern China-Myanmar border, the representative malaria epidemic area in Asia [25], [28]. In this study, 93 out of 130 different DBL1α transcripts identified in 9 field isolates of clinical adult patients (most were of mild symptoms) from Yunnan-Myanmar border were novel compared with those of other areas, suggesting a restricted local *var* gene repertoire in this area with limited overlap to other populations. This observation points to the existence of geographic population structures in various areas as previously observed by analysis of microsatellite or single nucleotide polymorphism [22], [42]. Structure feature analysis of the DBL1α sequences in terms of the conserved cysteine motif revealed a major distribution of four-cysteines subtypes (∼75%) reflective of a relatively lower virulence in these clinical isolates. Recent studies reported that the expression of different *var* gene types was under selection of host immune pressure [21], [43], [44], [45]. Unlike the non-immune volunteers or severe malaria in children investigated in other studies, the symptomatic malaria adult patients from Yunnan area had undergone multiple infections in past years suggesting that previous exposure afforded a measure of natural immunity.

Although there are some inconsistency among those studies on the association between *var* gene expression and severity of malaria disease investigated in several geographical isolates from Africa, it is recognized that the expression of *upsA*- and partial *upsB* (*B*/*A*)-subtype *var* genes correlates to the severe malaria disease [15], [16], [18], [46], [47]. Nevertheless, the underlying mechanism is far from being understood. In the present study, through sequence similarity analysis of DBL1α domain, *upsB* and *upsC var*s were appeared to be overlapping, however, *upsA var*s could be distinguished from other subtypes. It was therefore feasible to investigate the possible link between *upsA var*s and parasite virulence. Interestingly, we found that the *upsA var*s transcription had a potential link to the higher parasitemia of isolates in peripheral blood by different strategies (cDNA cloing/sequencing and qRT-PCR). Since these samples are collected from those patients under the same criteria including the symptom, age, sampling location and time point after the occurrence of disease, infection history, etc., it is likely that the expression of group A PfEMP1 confers the more effective growth rate on the parasites, probably due to better sequestration to avoid the clearance by the host immune system. This point is supported by another independent study with *ex vivo* samples which showed that NF54 parasites expressing group A and B *var*s had expanded more effectively *in vivo* compared to those expressing other *var* genes in experimentally infected humans [48]. Therefore, this finding suggests A-type PfEMP1 variant-mediated high parasitemia may contribute to the virulence of parasites in patients. However, since the difference of parasitemia in patients might be due to some other factors (patient genetic background, infection history, and parasite factors), the dominant expression of *upsA vars* would not be the only determinant to control the growth rate of parasites in natural infection. As we observed, the isolate F08B-33 exhibited a dominant transcription of *upsA vars* as well as ICAM-I-binding activities, however, it did not show a significantly higher parasitemia compared with other isolates. Hence, further experiments using more malaria isolates with various severities are needed to confirm the correlation between *in vivo* growth rate and *var* gene expression pattern (transcription and protein level) and the underlying mechanism. In addition, dynamic transcription analysis by qRT-PCR at different developmental stages in individual cycles revealed a link between the ICAM-1 binding phenotype and transcription of *upsA var*s, which supports the previously observed association between *upsA*-subtype *var*s and severe malaria since ICAM-1-mediated cytoadhesion has been implicated in pathogenesis of severe malaria [49], [50], [51].

In a homogeneous laboratory *P.falciparum* clone selected by a monoclonal antibody against a specific PfEMP1 or a unique receptor as CSA, it has been generally accepted that only one *var* gene is dominantly transcribed throughout the blood-stage cycle, and translated into the specific PfEMP1 protein which is exported onto the surface of the iRBC and mediates the cytoadhesion phenotype of the culture [30], [52], [53]. In other words, the dominantly transcribed *var* gene in a single cycle is expected to be translated into the PfEMP1 mediating a specific phenotype. However, due to the qRT-PCR technique with subtype-specific *var* primers for *var* transcription profiling had been validated only in rings, the transcriptional dominance order among various *var*s in trophozoites might not reflect the actual status. It has also been demonstrated that *var* transcriptional levels in trophozoites are 10–100 times lower than that in rings [53], resulting in very dubious quantification of transcript levels in the trophozoite states. This is contrary to our transcription data where the *upsBC1* level was almost similar between rings and trophozoites. Considering that the *upsBC1* and *BC2* primer pairs are targeting the exon 2 region of *var* genes [15], it is likely that these high transcriptional signals are from sterile *var* transcripts due to the intron promoter activity as reported previously [33], [54]. While the transcription level of the active genes in trophozoites are much lower compared with rings, the quantification of *var* gene transcription profile in this stage might be biased by the sterile transcripts. Therefore, since the PfEMP1 product appeared on the iRBC surface at ∼16 hr post invasion [52], it is rational to speculate the dominant *var* transcripts in rings, including the *uspA var*s, would be responsible for the cytoadhesion phenotypes in the late stage, which is consistent with our observation that the higher transcription level of *uspA var*s in rings is linked to the higher parasitemia *in vivo* and ICAM-1-binding *in vitro*.

The potential turn over of *var*s expression pattern in culture *in vitro* was considered as an obstacle in evaluating their functions. A recent study with “*ex vivo*” 3D7 samples had shown a rapid down-regulation of overall transcription level of *var* genes at ring stage, especially the *upsA*, -*D* and -*E* had a significantly faster rate of transcriptional reduction in the first ∼10 days of culture [27]. It is worthy of note that the dominant transcripts throughout the cultivation duration in their samples were only *upsB var*s. In addition, the transcription profiling in trophozoites were not performed. In the present study, transcription measurement of *var* genes at both ring and trophoziote stages over the course of ∼2 month post-thaw did undergo a rapid turn over event for *upsA* subset as well as *upsB/A var*s in the second cycle, indicating the virulence-related *var* subtype are more sensitive to the absence of immune pressure or other signals in host during the first cycle *in vitro*. Surprisingly, the declined *var*s were up-regulated again immediately after the fourth cycle in culture, which was not observed in the previous “*ex vivo*” 3D7 samples. It seems that the naturally infected isolates are able to adapt to the *in vitro* culture condition more rapidly than the parasites of passive infection.

It is well known that the CD36-binding phenotype was the most common in cultivated field isolates from patients with various symptoms, and group B and C *var* genes encode CD36-binding PfEMP1 variants in 3D7 strain [36], [55]. Similarly, the cytoadherent phenotypes of the cultivated field isolates from patients with symptomatic malaria in the Yunnan area in our study maintained the CD36 receptor-binding activity as the major phenotype during the ∼2 months of culture. However, it is worthy of note that the initial phenotype at collection time point might be different, e.g. the apparent ICAM-1 binding of F08B33 isolate during the first week in culture (Figure 6). This suggests an expression switching event among PfEMP1 variants occurred rapidly during the initial adaptive cultivation *in vitro* even though no apparent difference of *var* transcription in trophozoites was observed, which may be also linked to the role of *upsA var* transcripts in rings.

In summary, we conducted a systematic analysis of sequence diversity of the *var* repertoire, a dynamic transcription profile of the different subsets of *var* genes, and a cytoadherence analysis of parasites from malaria patients during a adaptive cultivation *in vitro*. Our results showed a restricted *var*-DBL1αrepertoire in this area, and a greater proportion of *var* genes containing a Cys4 DBL1α domain than seen in similar studies in Africa. This work extends the global database of *var* gene repertoire in various geographic isolates. Further, our data raised a new role of the ring-stage transcribed subset of *var* genes (*upsA* subtype) in development of malaria parasite in patients. Here we suggested the A-subtype PfEMP1 variants might be linked to the rapid growth rate of isolates *in vivo*. Finally, it should be cautious in interpreting the experimental data and understand the nature of *var* gene family from the *in vitro* cultivated parasites due to the observation of a rapid switching of *var* transcription and cytoadherent phenotype of field isolates during the initial cultivation process.

## Materials and Methods

### Ethics Statement

In this study, patients who sought medical care at the local Center for Disease Control and Prevention (CDC) in Yunnan from 2006 to 2008 were recruited with written informed consent, and the clinical protocol was approved by the Internal Review Board of Second Military Medical University, China.

### Culture of *Plasmodium falciparum* laboratory line FCC1/HN

*Plasmodium falciparum* FCC1/HN line was isolated from Hainan Island, China, established as a laboratory culture in 1979 and subsequently adapted to cultivation in RPMI 1640 medium (Invitrogen) containing 25 mM Hepes, 2 mM L-glutamine, and supplemented with 0.1 mM hypoxanthine (Sigma), 20 µg/ml gentamicin (Sigma), 15% rabbit serum and 2–4% (v/v) type O^+^ erythrocytes under the condition of 5% CO_2_ at 37°C [56], [57].

### *Plasmodium falciparum* isolate samples

The malaria patients participating in this study were residents in the border area of south China (Yunnan province) and Myanmar. Malaria in this area is subject to seasonal variation with an increased incidence level occurring from July to September. Adult male farmers are at higher risk for *P. falciparum* infection than the rest of the population because their occupation leads to exposure to *Anopheles minimus*, a potent malaria vector. Blood samples came from patients who sought medical care at the local Center for Disease Control and Prevention (CDC) in Yunnan from 2006 to 2008. Malaria diagnosis was done by microscopic examination of blood smears. Parasitemia was measured relative to leukocyte numbers per µl of blood, with an average of 5000 leukocytes/µl of blood. In this study, we selected symptomatic malaria patients who were >5 years of age (most were adult). In general, they had *P. falciparum* in peripheral blood with parasitemia >1000 infected red blood cells (iRBC) per µl of blood, an axillary temperature <40°C, and a history of fever. Basically, patients with severe malaria, including cerebral and placental malaria, were treated immediately and hence excluded from this study except two samples with symptom between mild and severe malaria (YN3 and YN29 in Table 1). The sample size and the way of sample collection from patients were in accord with different analysis in this study:

Analysis 1: samples in this assay were used to analyze the sequence diversity of *var*-DBL1α domain by cDNA clone and sequencing. Hence, the fresh blood samples from patients were mixed with anticoagulant, EDTA and RNAlater solution, a RNA protection reagent (Ambion). Then the samples were frozen at −80°C until total RNA extraction. Totally 22 samples were collected for this aim, and 15 isolates were randomly selected for total RNA extraction. However, only 9 samples were analyzed for *var* gene diversity since others did not produced sufficient *var*-DBL1αsequences by cDNA cloning with total RNAs of poor quality (see Figure 3 and Table S1).

Analysis 2: isolates used in this assay were aimed to measure the dynamic *var* gene transcription patterns both at ring- and trophozoite-stage, and cytoadhesion assay over time during the adaptive cultivation of ∼2 months *in vitro* (shown in Figure 4A). Hence, fresh blood samples were cryopreserved in glycerolyte 57 (Baxter), and frozen in liquid nitrogen. Totally 35 isolates were collected for this assay, and the actual number of samples used in this assay was described in the result section. We attempted to cultivate 10 field isolates for assay 1 whereas 13 isolates for assay 2 (Figure 4A), which were randomly selected to be thawed. Among these samples, 6 isolates (assay 1) and 8 isolates (assay 2) reached ∼1% parasitemia within in the first 5–10 days and subsequently adapted the growth condition *in vitro* (see Table 1 and S1).

### Cultivation of patient isolates *in vitro*

Frozen isolates from malaria patients were thawed for analysis as described previously [44], and cultured in the RPMI 1640 medium (Invitrogen) containing 25 mM Hepes, 2 mM L-glutamine, and supplemented with 0.1 mM hypoxanthine (Sigma), 20 µg/ml gentamicin (Sigma), 0.5% Albumax II, 2% human serum (type AB^+^). Cultures were grown in media with type O^+^ erythrocytes at a 3% hematocrit under 2% O_2_, 5.5% CO_2_, 92.5% N_2_ at 37°C [57]. The ring-stage parasites were synchronized with 5% sorbitol or Percoll alternatively.

### Total RNA extraction and complementary DNA (cDNA) synthesis for cloning

Total RNA was isolated both directly from frozen patient blood samples and from *in vitro* cultured samples. For the cultivated parasites, the rings of 10–20 hr or trophozoites of 25–35 hr post invasion were harvested, respectively. RNA was extracted with Trizol (Invitrogen) according to the manufacturer's instruction with modification as described elsewhere [58]. Subsequently, all of the RNA extracts were treated with DNase I (Ambion) to remove the potential contamination of genomic DNA. RT-PCR amplification, cloning and sequencing of *var*-DBL1α domain DBL1α sequences were amplified using the following primers: DBLαAF, 5′GCACG(A/C)AGTTT(C/T)GC3′, and DBLαBR, 5′GCCCATTC(G/C)TCGAACCA3′ [19]. Negative control reactions were performed in the same conditions without reverse transcriptase. DBLα1 sequences from each sample were isolated from a 50 µl PCR reaction containing 2 µl of cDNA template (32 cycles: 30 s at 94°C, 30 s at 42°C and 30 s at 65°C followed 5 min at 6 5°C). The amplified products of DBL1α amplification reactions were separated by electrophoresis in 1.5% agarose and the product extracted the gel (Qiagen). The purified DBL1α DNA fragments were cloned into a TA vector as described by the manufacturer (Promega). For each clinical isolate, we sequenced 50∼100 cDNA clones, and only those isolates with >50 sequence reads were used in our analysis.

### Sequence analysis

Sequences edited with DNAstar software (version 6) aligned in batches using ClustalW analysis (http://www.ebi.ac.uk/clustalw/) using the default settings (Gonnet250 matrix, gap opening penalty  = 10.0, gap extension penalty  = 0.2, gap closing penalty = −1, gap separation penalty = 4). Unrooted phylogenetic trees (based on amino acid sequences) were constructed by p-distance/neighbour-joining (NJ) method with 1000 bootstrap replicates using MEGA version 3 [59]. Observed clusters from each tree were confirmed visually on alignments.

### Validation of *var* subtype-specific qRT-PCR primers in Yunnan isolates

Primers specific for subtype or individual *var* genes were described previously [15], [60]. These *var* subtype-specific primes had been validated in 3D7 and southern Tanzania isolates. To validate these primers in the samples from Yunnan area, all primers were tested on genomic DNA from our isolates. The genomic DNAs were extracted with the GenElute™ Mammalian Genomic DNA Miniprep Kit (sigma). All primers had amplification efficiencies (E) between 1.80 and 2. The relative copy numbers of amplified subtype *var* genes by these primers were compared to the internal single-copy control gene, *Seryl-tRNA synthetase*. The amplification efficiency for field isolate gDNA was similar to that for 3D7 gDNA for *upsA1*, *A2*, *A3*, *B2*, *BC1*, *C2*, *Var1* (*upsD*) and *Var2* (*upsE*). Because there are overlapping members between *BC1* and *BC2*, *C1* and *C2*, respectively, we used the primers of *upsA2*, *B2*, *BC1*, *BC2*, *C1*, *C2*, *D* and *E* subgroup in this study.

### qRT-PCR

Parasite cultures were tightly synchronized by sorbitol lysis for total RNA extraction. Total RNA of synchronous parasite culture was extracted using Trizol reagent (Invitrogen) according to the protocol as described previously [58]. The potential genomic DNA contamination were removed by DNase I treatment with DNA-free kit (Ambion), and the resulting RNA samples were tested by PCR of 35 cycles with primers of the housekeeping gene seryl-tRNA synthetase (*PF07_0073*) to confirm the lack of genomic DNA contamination. 500 ng total RNA was subjected to reverse transcription reaction with a mixture of oligo dT and random hexamer primers as reverse primers in a 20 µl reaction according to the manufacture's recommendations (Invitrogen). Negative control reactions were performed in the same conditions without reverse transcriptase. All runs were performed using an Applied Biosystem 7300 detection system in a 20 µl reaction contained 0.5 µl cDNA, 1×SYBR Green I Mastermix, 0.2 µM specific primer pair for individual gene or var gene subtypes tested, and each reaction was run in duplicate. Seryl-tRNA synthetase was used as endogenous control gene. The qRT-PCR reaction conditions were as follows: 95°C for 10 s, followed by 40 cycles of 94°C for 20 s, 50°C for 20 s and 62°C for 20 s.

Transcripts were quantified as follows: the threshold cycle (*Ct*) is defined as the cycle number at which the quantity of fluorescence product passes a pre-determined threshold. The relative amounts were calculated using the equation: *ΔCt*  =  Ct_*var* subtype_ - Ct_Seryl-tRNA synthetase_. *ΔCt*s were then converted to relative copy numbers with the formula 2^-*ΔCt*^. Dissociation curves were generated and the homogeneity of the products were verified by observation banding pattern after electrophoretic separation of the reaction mix.

### Cytoadherence assays on immobilized receptors

Cytoadherence assays were carried out as described [61]. Briefly, host receptors including CD36, ICAM-1, CSA and Hyaluronan (HA) were used for panning of parasites. Plastic Petri dishes were coated overnight at 4°C with 1xPBS containing either 1 mg/ml CSA sodium salt from bovine trachea (Sigma), 100 µg/ml HA sodium salt from bovine vitreous humor (Sigma), 10 µg/ml recombinant human CD36 (R&D Systems), 10 µg/ml recombinant human ICAM-1 (R&D Systems) or 1% BSA. These receptors were spotted onto a 150 mm petri dish (BD Falcon) in duplicates and incubated in a humid box overnight at 4°C. Trophozoite-stage parasites (24–30 hr post-invasion) infected erythrocytes were enriched by Percoll and resuspended in adhesion medium containing RPMI 1640 and 25 mM Hepes, pH6.8. Binding assays were incubated at 37°C for 1 hr without agitation. Dishes were gently washed 3 times with adhesion medium and once with 1xPBS. The bound cells were fixed with 2% glutaraldehyde (Amresco) in PBS and stained with Giemsa solution (Gibco). The average numbers of parasites adherent to each receptor in duplicate spots (cells per mm^2^) were counted in 25 continuous fields by light microscopy.

### Statistic analysis

SPSS16.0 software was used to perform Mann-Whitney U test to evaluate the difference of parasitemia between *upsA* and other group. Difference was considered significant if P value was <0.05 (2-tailed). The distribution analysis of *var*-DBL1α sequences were were compared using *χ* ^2^ test. “*”: *p*<0.01 between tested group and control.

## Supporting Information

Figure S1 **Transcriptional pattern and cytoadherent features of unselected** ***P.falciparum*** **FCC1/HN line.** (A) Synchronized trophozoite-stage parasites were used to measure the transcriptional level of various subtype *var* genes. (B) Cytoadherence assay of FCC1/HN line with CD36, ICAM-1, CSA, and HA purified receptors with BSA as negative control.
(TIF)

Figure S2 **The ring-stage transcription profile of** ***var*** **gene family in LZF22 isolate.** The analysis was started after two weeks post-thaw based on the stuff amount. The *Seryl-tRNA synthetase* gene was used as the endogenous control. The time point when the *upsA var*s were dominantly transcribed is indicated by arrow.
(TIF)

Table S1 Clinical assessment of symptomatic malaria patients recruited in this study (supplementary to Table 1).
(DOC)

Table S2 *Plasmodium falciparum* DBL1α contigs of *var* transcripts in field isolates from symptomatic patients in Yunnan-Myanmar area.
(DOC)

Table S3 Genotyping of six isolates during the period of adaptive cultivation with microsatellite assay (linked to Figure 4B).
(DOC)
