# Supplementary figures and images for: From In Vivo to In Vitro: Dynamic Analysis of Plasmodium falciparum var Gene Expression Patterns of Patient Isolates during Adaptation to Culture
Source: PLoS One. 2011 Jun 6;6(6):e20591. doi: 10.1371/journal.pone.0020591 (PMC3108956; doi:10.1371/journal.pone.0020591)

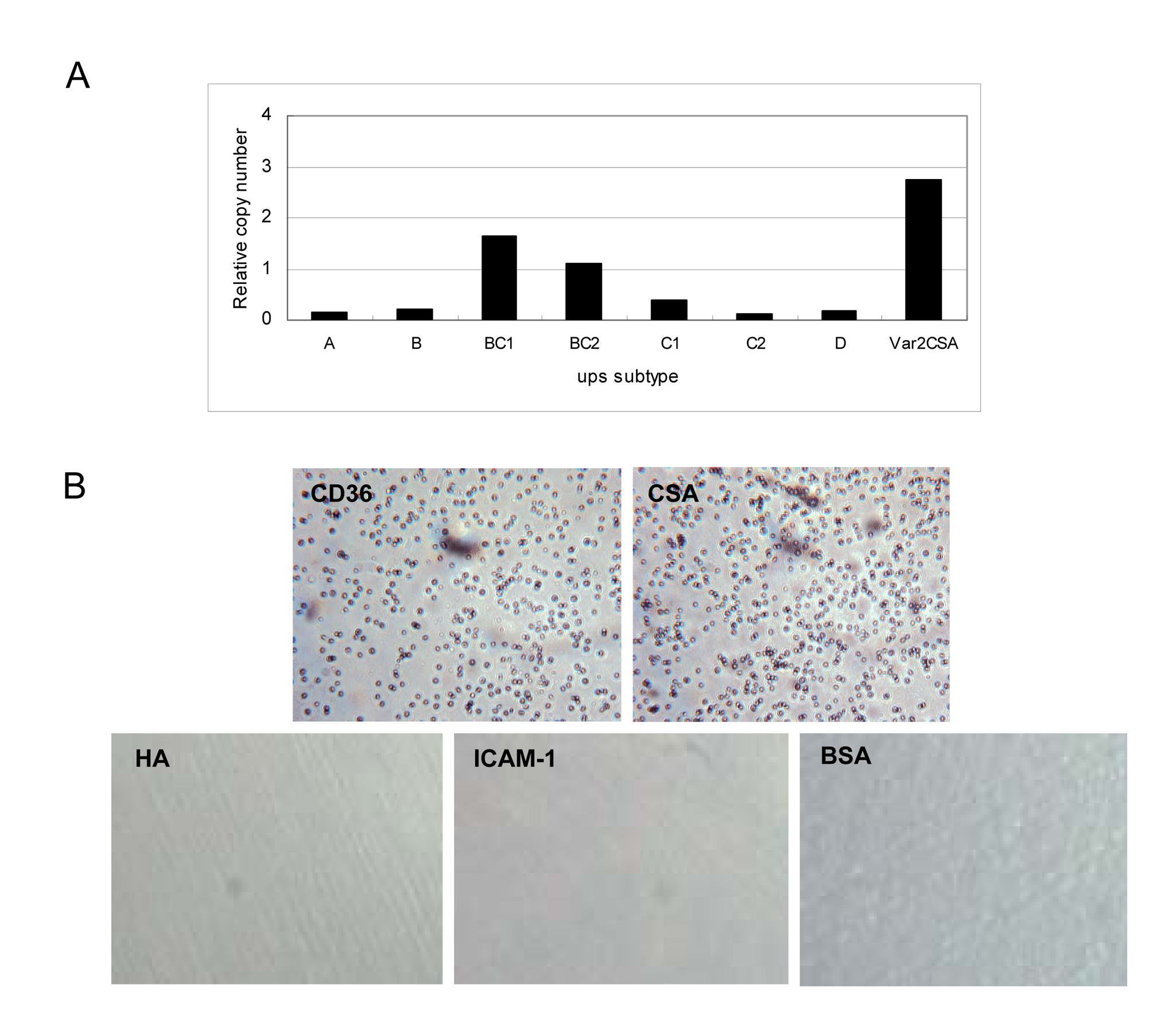

Supplement: Figure S1 — Transcriptional pattern and cytoadherent features of unselected P.falciparum FCC1/HN line. (A) Synchronized trophozoite-stage parasites were used to measure the transcriptional level of various subtype var genes. (B) Cytoadherence assay of FCC1/HN line with CD36, ICAM-1, CSA, and HA purified receptors with BSA as negative control. (TIF) [file pone.0020591.s001.tif]

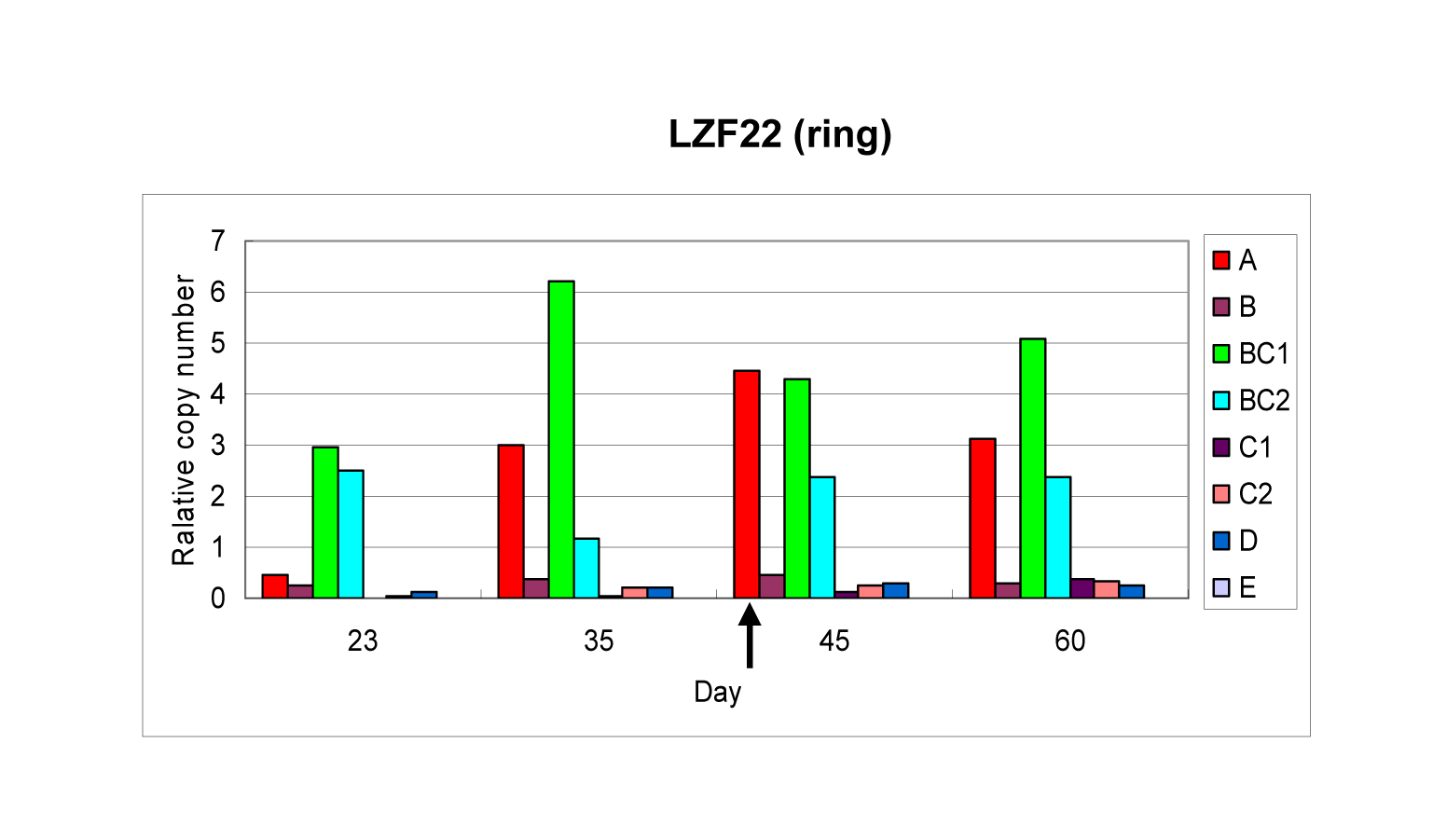

Supplement: Figure S2 — The ring-stage transcription profile of var gene family in LZF22 isolate. The analysis was started after two weeks post-thaw based on the stuff amount. The Seryl-tRNA synthetase gene was used as the endogenous control. The time point when the upsA vars were dominantly transcribed is indicated by arrow. (TIF) [file pone.0020591.s002.tif]
