# Supplementary material for: From In Vivo to In Vitro: Dynamic Analysis of Plasmodium falciparum var Gene Expression Patterns of Patient Isolates during Adaptation to Culture
Source: PLoS One. 2011 Jun 6;6(6):e20591. doi: 10.1371/journal.pone.0020591 (PMC3108956; doi:10.1371/journal.pone.0020591)
Supplement: Table S1 — Clinical assessment of symptomatic malaria patients recruited in this study (supplementary to Table 1). (DOC) [file pone.0020591.s003.doc]

**Table S1.** Clinical assessment of symptomatic malaria patients recruited in this study (supplementary to Table 1)

| **Isolates** | **Address** | **Infection history in the year** | **Sampling timepoints (day)** | **Number of cloned DBL1α tags** | **Number of DBL1α contigs** |
| --- | --- | --- | --- | --- | --- |
| A2 | Lianghe | + | 5 | 51 | 16 |
| A3 | Tengchong | + | 1 | 59 | 16 |
| A7 | Dali | + | 2 | 53 | 7 |
| A15 | Tengchong | + | 4 | 58 | 18 |
| A16 | Tengchong | + | 3 | 55 | 15 |
| A19 | Tengchong | + | 4 | 67 | 18 |
| A20 | Tengchong | + | 3 | 56 | 21 |
| A21 | Ruili | + | 6 | 68 | 14 |
| A22 | Tengchong | + | 6 | 60 | 21 |
| F07-4 | Dehong | + | 14 | ND | ND |
| F08B-5 | Dehong | - | 7 | ND | ND |
| F08B-9 | Dehong | + | 3 | ND | ND |
| F08B-33 | Dehong | + | 9 | ND | ND |
| F08B-34 | Dehong | + | 7 | ND | ND |
| LZF22 | Laza | + | 1 | ND | ND |
| LZF25 | Laza | + | 3 | ND | ND |
| LZF26 | Laza | + | 8 | ND | ND |
| YN3 | Laza | + | 4 | ND | ND |
| YN8 | Laza | + | 6 | ND | ND |
| YN11 | Laza | + | 2 | ND | ND |
| YN27 | Laza | + | 4 | ND | ND |
| YN29 | Laza | + | 3 | ND | ND |
| YN53 | Laza | + | 15 | ND | ND |

Note: “ND” not done.
