# Supplementary material for: From In Vivo to In Vitro: Dynamic Analysis of Plasmodium falciparum var Gene Expression Patterns of Patient Isolates during Adaptation to Culture
Source: PLoS One. 2011 Jun 6;6(6):e20591. doi: 10.1371/journal.pone.0020591 (PMC3108956; doi:10.1371/journal.pone.0020591)
Supplement: Table S2 — Plasmodium falciparum DBL1α contigs of var transcripts in field isolates from symptomatic patients in Yunnan-Myanmar area. (DOC) [file pone.0020591.s004.doc]

**Table S2．***Plasmodium falciparum* DBL1α contigs of *var* transcripts in field isolates from symptomatic patients in Yunnan-Myanmar area.

| **DBL1**α | **group** | **PoLV1-PoLV2-PoLV3-(cys)n-PoLV4-length** |
| --- | --- | --- |
| *A16-D49** | 2 | LFLG-VREY-KALT-2-LTNL-114 |
| *A21-38* | 3 | LFLG-VREA-KALT-2-LTNL-114 |
| A22-F52 | 2 | LYLG-IREY-KALT-2-LTNL-114 |
| *A16-D50* | 3 | MFLG-LRED-KALT-2-LTNL-136 |
| *A15-g95* | 2 | MFLG-VREY-KALT-2-ITNL-113 |
| *A22-21* | 2 | LFLG-VREY-DALT-2-LTNL-111 |
| *A22-X47* | 2 | LFLG-IREY-EALT-2-LTNL-111 |
| *A20-D4* | 2 | LYLG-VREY-EALT-2-PTNL-110 |
| A20-F20 | 3 | LYLG-LREH-KALT-2-LTNL-118 |
| *A22-F41* | 3 | LYLS-LRED-KAIT-2-PTNL-131 |
| *A22-F25* | 4 | LYRG-LRED-EALT-4-PTNF-125 |
| A21-20 | 4 | LYLG-LRED-KALT-4-PTYF-125 |
| A22-F31 | 4 | LFRG-LRED-KAIT-4-PTYF-122 |
| *A15-4-27* | 4 | LYRG-LRED-KAIT-4-PTYF-122 |
| *A19-30* | 4 | LYRG-LRED-EALT-4-PTYF-131 |
| *A16-D57* | 4 | LYSG-LRED-KALT-4-PTYF-136 |
| *A20-E19* | 4 | LFYG-LRED-KALT-4-PTYF-128 |
| *A19-k17* | 4 | LYGG-LRED-DAIT-4-PTYF-119 |
| *A22-F4* | 4 | LFHG-LRED-YAIT-4-PTYF-125 |
| *A3-E37* | 4 | LYRR-LRED-YAIT-4-PTYF-125 |
| *A22-40* | 4 | LYRR-LRED-KALI-4-PTYF-122 |
| *A19-6* | 4 | LYVG-LRED-KAIT-4-PTYF-126 |
| *A19-k39* | 4 | LYRG-LRED-KAIT-4-PTYF-117 |
| *A19-k1* | 4 | LYRG-LRED-YAMT-4-PTYF-119 |
| *A3-E14* | 4 | LYRR-LRED-KAIT-4-PTYF-123 |
| *A2-F24* | 4 | LYRR-LRED-KAIT-4-PTYF-125 |
| *A3-E32* | 4 | LYRR-LRED-KALT-4-PTYF-133 |
| A7-D35 | 4 | LYLG-LRED-KAIT-4-PTYF-128 |
| *A16-D30* | 4 | LYRR-LRED-KALT-4-PTYF-134 |
| A13-G32 | 4 | LYRG-LRED-KAIT-4-PTYF-115 |
| A7-K6 | 2 | LFLG-IREY-KAIT-2-LTNL-112 |
| A15-g26 | 2 | LFLG-LREY-KAIT-2-PTNL-108 |
| *A2-E14* | 2 | LFLG-VREY-KAIT-2-PTYF-109 |
| *A3-F14* | 2 | LYLH-FREY-KAIT-2-PTYL-111 |
| A22-3 | 2 | LYLD-LREY-KAIT-2-PTNL-108 |
| A20-D6 | 2 | LFLG-VREY-KAIT-2-PTNL-109 |
| *A15-4-40* | 5 | LFYG-VREY-KAIT-4-PTYF-122 |
| *A15-g97* | 5 | LFLG-VREY-KAMI-4-PTYF-109 |
| *A15-g99* | 5 | LFLG-LREY-KALT-4-PTYF-112 |
| *A2-F7* | 5 | LYLG-IREY-KAIT-4-PTYF-114 |
| *A16-D58* | 4 | LYLG-LRED-KAIT-4-PTYF-131 |
| *A3-E33* | 4 | LFLG-LRED-EAIT-4-PTYF-121 |
| A19-k5 | 4 | LYRG-LRED-EAIT-4-PTYF-122 |
| A7-g4 | 4 | LYSG-LRED-KAIT-4-PTYF-121 |
| *A2-F13* | 4 | LYLG-LRED-RAIT-4-PTYF-121 |
| *A3-D2* | 4 | LYSG-LRED-KAIT-4-PTYF-132 |
| *A7-D34* | 4 | LYGG-LRED-EAIT-4-PTFF-130 |
| *A3-E45* | 4 | LYRG-LRDD-KAIT-4-PTYF-139 |
| A15-G38 | 4 | LYLG-LRED-KALT-4-PTYF-124 |
| A16-D38 | 4 | LYLG-LRED-KALT-4-PTYF-128 |
| *A16-D59* | 4 | LYLG-LRED-KAIT-4-PTNF-122 |
| *A3-E1* | 5 | LYLR-IREY-KAIT-4-PTNF-108 |
| A22-27 | 4 | LFLG-LRED-KAIT-4-PTYF-127 |
| A2-F10 | 4 | LFLG-LRED-KALT-4-PTYF-123 |
| *A21-17* | 4 | LYGG-LRED-KALT-4-PTYF-137 |
| A19-28 | 4 | LYLG-LRED-YAIT-4-PTYF-121 |
| *A19-k26* | 4 | LFHG-LRED-EAIT-4-PTYF-129 |
| *A2-D30* | 4 | LYRG-LRED-EAIT-4-PTYF-130 |
| A22-38 | 4 | LYLG-LRED-KALT-4-PTYF-126 |
| *A16-D100* | 5 | LYLG-LREY-KAIT-4-PTYF-134 |
| *A16-D104* | 4 | LYRG-LRED-KAIT-4-PTYF-127 |
| A21-37 | 4 | LYLG-LRED-KAIT-4-PTYF-125 |
| A21-k27 | 4 | LYLG-LRED-KAIT-4-PTYF-126 |
| A21-23 | 4 | LYLG-LRED-EALT-4-PTYF-128 |
| *A22-F32* | 4 | LYLG-LRED-EALT-4-PTYF-126 |
| *A3-F10* | 4 | LYLG-LRED-KAIT-4-PTYF-136 |
| *A2-F45* | 4 | LYLG-LRED-KAIT-4-PTYF-133 |
| *A22-17* | 4 | LYLG-LRED-EAIT-4-PTYF-132 |
| *A19-k40* | 4 | LFYG-LRED-KALT-4-PTYF-129 |
| *A20-E28* | 4 | IFRG-LRED-IAMT-4-PTYF-122 |
| *A20-D26* | 4 | MFLG-LRED-KAMT-4-PTYF-123 |
| *A21-k3* | 4 | LFYG-LRED-EALT-4-PTFL-128 |
| *A19-25* | 4 | LFLG-LRED-EALT-4-PTYF-138 |
| *A3-E10* | 4 | LFLG-LRED-KAIT-4-PTYF-132 |
| *A16-D53* | 4 | LFLG-LRED-EAIT-4-PTYF-128 |
| A3-D5 | 4 | LFYG-LRED-EALT-4-PTYF-118 |
| *A20-D30* | 4 | LFYG-LRKD-KALT-4-PTYF-127 |
| *A21-28* | 4 | LYLG-LRED-EAIT-4-PTYF-121 |
| *A2-F36* | 4 | LFLG-LRED-EALT-4-PTYF-137 |
| *A3-F5* | 4 | LYLG-LRED-EAIT-4-PTYF-133 |
| *F46* | 4 | LYLG-LRED-KAIT-4-PTYF-138 |
| *A13-G4* | 6 | LYLG-LRED-KAIT-5-PTYF-124 |
| *A3-F8* | 4 | LYRR-LRED-KAIT-4-PTYF-119 |
| *A16-D36* | 4 | LFLG-LRED-KAIT-4-PTYF-131 |
| *A19-k41* | 4 | LYRG-LRED-KAIT-4-PTYF-129 |
| A20-E18 | 4 | LYLG-LRED-KALT-4-PTYF-128 |
| *A22-F12* | 4 | LYIR-LRED-KAIT-4-PTNF-117 |
| *A16-D77* | 4 | LYIR-LRND-KAIT-4-PTYF-125 |
| *A22-F29* | 4 | LYIR-LRED-EAIT-4-PTYF-120 |
| *A19-21* | 4 | LFIG-LRED-EAIT-4-PTNL-126 |
| *A21-12* | 4 | LFIG-LRED-DAIT-4-PTYM-128 |
| A15-g122 | 4 | LFIG-LRED-KAIT-4-PTYF-123 |
| *A16-D105* | 4 | LYIR-LRED-EAIT-4-PTYF-115 |
| *A13-g12x* | 4 | LFIG-LRED-TAIT-4-PTYF-124 |
| *A22-F50* | 4 | LYLG-LRED-KAIT-4-PTYF-137 |
| A2-F3 | 4 | LFIG-LRED-KAIT-4-PTYF-126 |
| A19-k42 | 5 | LFLG-LREY-NAMI-4-LTNF-107 |
| *A2-E36* | 4 | LYRR-LRED-NSII-4-LTNF-123 |
| A21-k5 | 3 | LYLG-LRED-KAIT-2-PTYL-121 |
| *A20-D29* | 4 | LFLG-LRND-KAIT-4-PTNL-121 |
| *A7-K5* | 3 | LYLG-LRED-KAIT-2-TTYF-121 |
| *A20-D9* | 2 | LYLG-LREY-KAIT-2-PTNL-117 |
| *A20-F8* | 3 | LYLG-LRED-KAII-2-PTNF-126 |
| *A7-K82-T7* | 3 | LYRG-LRED-DAIT-2-PTNF-119 |
| A13-G116 | 3 | LYLG-LRED-KAIT-2-PTNF-115 |
| *A16-D117* | 3 | LYRG-LRED-KAIT-2-PTNF-117 |
| *A15-g100* | 6 | LYLG-LRED-EAIT-3-LTNF-119 |
| A3-D23 | 4 | LYIG-LRED-KALT-4-PTYF-126 |
| A19-k34 | 4 | LFLG-LRED-NAIT-4-LTNF-116 |
| *A19-49x* | 4 | LYLG-LRED-KAMT-4-PTNL-122 |
| *A21-k24* | 4 | LFLG-LREH-KAMI-4-PTNL-120 |
| *A3-E31* | 4 | LYGR-LRED-EAIT-4-PTYF-127 |
| A22-11 | 4 | LYLG-LRED-EAMT-4-PTYF-117 |
| *A20-D13* | 4 | LYRR-LRED-KAIT-4-YVPQ-118 |
| A22-33 | 4 | LFIG-LRED-KAIT-4-PTYF-120 |
| *A22-37* | 4 | LYRG-LRED-KAMT-4-PTYF-115 |
| *A2-F48* | 1 | MFKP-LRED-KAIT-2-LTNL-117 |
| A13-G27 | 1 | MFKP-LRED-RAIT-2-LTNL-117 |
| A15-5-14 | 3 | MFRS-LREA-EAIT-2-PTNL-115 |
| *A15-5-8* | 1 | MFKS-LRED-KAIT-2-PTNL-113 |
| *A20-F18* | 1 | MFKR-LREA-EAIT-2-PTNL-117 |
| *A7-K29* | 1 | MFKR-LRND-KAIT-2-PTNL-116 |
| A19-17 | 1 | MFKR-LRED-KAIT-2-PTYL-117 |
| A20-D38 | 1 | MFKS-LREA-KAIT-2-PTYL-115 |
| A20-F12 | 1 | MFKS-LREA-KAIT-2-PTYL-115 |
| A15-g128 | 1 | MFKS-LREA-RAIT-2-PTYL-115 |
| *A8g8* | 1 | MFKR-LRED-RAIT-2-PTNL-116 |
| *A21-k35* | 3 | MWDQ-LRSD-EAMQ-2-TPYD-94 |
| *A3-F19* | 4 | MFKS-LREA-EAIT-4-PTYF-115 |
| SR-80 | 1 | MFKP-LREA-EAIT-2-PTNL-115 |

**Note: “***” *Var*-DBL1α sequences highlighted in italic represent the unique sequences of Yunnan field isolates compared with that of previously reported in other area.
