# Supplementary material for: From In Vivo to In Vitro: Dynamic Analysis of Plasmodium falciparum var Gene Expression Patterns of Patient Isolates during Adaptation to Culture
Source: PLoS One. 2011 Jun 6;6(6):e20591. doi: 10.1371/journal.pone.0020591 (PMC3108956; doi:10.1371/journal.pone.0020591)
Supplement: Table S3 — Genotyping of six isolates during the period of adaptive cultivation with microsatellite assay (linked to Figure 4B). (DOC) [file pone.0020591.s005.doc]

**Table S3. Genotyping of six isolates during the period of adaptive cultivation with microsatellite assay (linked to Figure 4B)**

| Time point *in vitro* | YN3 | YN8 | YN11 | YN27 | YN29 | YN53 |
| --- | --- | --- | --- | --- | --- | --- |
| 0 hr | 1* | 1 | 2 | 2 | 1 | 1 |
| 8 d | 1 | 1 | 1 | 1 | 1 | 1 |
| 16 d | 1 | 1 | 1 | 1 | 1 | 1 |

Note: ”*” number of sub-clones in each isolate at various time points from collection time (0 hr) to culture *in vitro*.
